# Erection hardness score or penile Doppler ultrasound: which is a better predictor of failure of nonsurgical treatment of erectile dysfunction?

**DOI:** 10.1093/sexmed/qfad009

**Published:** 2023-03-21

**Authors:** Alberto Costa Silva, Carlos Martins Silva, Afonso Morgado

**Affiliations:** Serviço de Urologia, Centro Hospitalar Universitário São João, 4200-319, Porto, Portugal; Serviço de Urologia, Centro Hospitalar Universitário São João, 4200-319, Porto, Portugal; Faculdade de Medicina da Universidade do Porto, 4200-319, Porto, Portugal; Serviço de Urologia, Centro Hospitalar Universitário São João, 4200-319, Porto, Portugal

**Keywords:** penile Doppler ultrasound, intracavernous injection test, erectile dysfunction, refractory erectile dysfunction, Erection Hardness Score

## Abstract

**Background:**

In the evaluation of men presenting for erectile dysfunction (ED), specific diagnostic tests, such as an intracavernous injection test (IIT) with Erection Hardness Score (EHS) assessment or penile Doppler ultrasound (PDU), may be necessary.

**Aim:**

The study sought to compare the prognostic value of PDU parameters with erection rigidity with EHS during IIT in predicting refractory ED after 5 years.

**Methods:**

Patients referred for ED were evaluated and had a PDU with at least 15 μg of intracavernous alprostadil and without any sexual stimulation. At 5 years of follow-up, current and past ED treatments were noted. Refractory ED was defined as having a penile prosthesis (PP) implanted, having failed nonsurgical treatments but having refused PP implantation, or having discontinuation of nonsurgical treatments due to loss of efficacy. Patients with hypogonadism and pelvic surgery were excluded. Receiver-operating characteristic curves were drawn and the area under the curve (AUC) was calculated.

**Outcomes:**

The outcome was the AUC for predicting refractory ED.

**Results:**

At 5 years, 69 men were still in follow-up with a mean age of 58.47 ± 10.39 years, and 13 (18.8%) were classified as having refractory ED. The AUC for the EHS, peak systolic velocity, end-diastolic flow, and resistive index to discriminate refractory ED were 0.820, 0.613, 0.730, and 0.714, respectively.

**Clinical Implications:**

EHS can be a good predictor of response to nonsurgical treatments in ED.

**Strengths and Limitations:**

This was a prospective study to compare IIT with PDU, and validated disease-specific questionnaires were used to assess both clinical efficacy and satisfaction. PDU was performed by a blinded third party. However, resulting from a single-center study, our sample size can be considered small, and the number of events observed was also low.

**Conclusion:**

Our data suggest that an abnormal EHS during an IIT is, at least, noninferior than an abnormal PDU in predicting those patients that will not respond to nonsurgical treatments and that will need a PP in long-term.

## Introduction

Erectile dysfunction (ED) is a common age-related condition, being one of the most prevalent over 60 years of age.^1–3^

More than one-third of ED patients do not respond to nonsurgical treatments even after optimization. These patients with refractory ED will probably need to have a penile prosthesis (PP) implanted to treat ED, and early recognition may help the approach.^4–6^

In the evaluation of men presenting for ED, evaluation tests like a penile Doppler ultrasound (PDU) or, sometimes, an intracavernous injection test (IIT) may be needed.^7^ Common PDU measurements, such as peak systolic velocity (PSV), end-diastolic flow (EDF), and resistive index (RI), can add quantifiable information to the IIT and may point to a clear etiology of vasculogenic ED.^8–11^ However, regardless of the etiology identified, most patients will follow the same treatment algorithms.^8^^,^^12^

The IIT is a straightforward diagnostic test that does not require an ultrasound machine or any Doppler measurements, and the Erection Hardness Score (EHS) can be easily applied.^13^^,^^14^ The EHS is a short inquiry that can accurately predict successful sexual intercourse with oral sildenafil citrate and intracavernous alprostadil in patients presenting for ED.^15^

In the recent past, a PDU’s added value was doubted, and it was shown that IIT may well suffice, if not exceed, a PDU as a predictor of response to ED therapy with oral sildenafil citrate. The need to perform a PDU instead of a more pragmatic IIT was questioned, as common penile Doppler measurements added no prognostic value.^16^ However, gauging only short-term endpoints may be misleading, and long-term results are often left unreported.

In that sense, have the EHS and the area under the curve (AUC), sensitivity, and specificity than the PDU in prediction failure of nonsurgical treatment?

## Methods

After approval by the hospital’s ethical committee, and individual consent was obtained, 200 consecutive men referred for ED by general practitioners between January 2016 and December 2018 were screened with a full medical and sexual history. Patients with the following conditions were excluded: unconfirmed diagnosis of ED, hypogonadism, Peyronie’s disease, contraindication for ED treatment with sildenafil or alprostadil, history of prostate cancer, radical pelvic surgery, penile surgery other than circumcision, and abnormalities found in PDU (shunts, intercavernous anastomoses, cavernous artery hypoplasia).

All patients had a PDU with 15 μg of IIT with alprostadil performed by a blinded third party. For patients who did not achieve their best-quality erection, alprostadil redosing with 15 μg or a second PDU were allowed. No visual sexual aid was used. Normal values for PDU were defined as the highest PSV >30 cm/s, an EDF lower than 3 cm/s, and an RI higher than 0.8.^17^ Bilateral measurements were made, but only the highest PSV and lowest EDF were used.

Erection rigidity was assessed during PDU at maximal erection point using the EHS and classified into 5 grades: 0 = penis does not enlarge, 1 = penis enlarges but is not hard, 2 = penis is hard but not hard enough for penetration, 3 = penis is hard enough for penetration but not completely rigid, and 4 = penis is completely hard and fully rigid.^18^ An abnormal EHS was defined as a score equal to or lower than 2.

All the patients were started on sildenafil citrate 100 mg and then underwent follow-up at 3 and 6 months, and thereafter at least yearly until the 5-year mark was reached. The option of PP implantation was reassured in every consult. At 5-year follow-up, patients completed the simplified International Index of Erectile Function (IIEF-5) questionnaire validated in Portuguese.^19^^,^^20^ Current and past ED treatments were noted, and if treatment was stopped, the main reason for stopping was also noted. If patients died, could not continue sexual activity, or were lost to follow-up, the last known treatment status was also noted.

Refractory ED at 5 years of follow-up was pragmatically defined as having a PP implanted, having failed nonsurgical treatments but refused PP implantation, or having discontinuation of nonsurgical treatments due to loss of efficacy.

### Statistical analysis

Data were collected and assessed using SPSS version 24 (IBM). The Shapiro-Wilk test was used to assess parameter distribution. All continuous variables with normal distribution are expressed as the mean ± SD, and non-normally distributed variables are expressed as the median (interquartile range [IQR]). Receiver-operating characteristic (ROC) curves were drawn for PSV, EDF, RI, and EHS using refractory ED at 5-year follow-up as the dichotomic variable, and AUCs were calculated. For AUCs <0.5, between 0.5 and 0.7, between 0.7 and 0.8, and >0.8, the test was considered worthless, acceptable, good, or excellent, respectively. DeLong’s empirical method was used to compare the AUCs in a pairwise approach. All tests were 2-sided, and statistical significance was considered at a *P* value <.05.

## Results

A total of 77 patients were included, and at 5 years of follow-up, 69 (89.62%) were still in follow-up, 3 (3.89%) were lost to follow-up, and 5 (6.49%) had died.

The patients’ mean age at 5 years of follow-up was 58.47 ± 10.39 years, and cardiovascular risk factors were common among the participants, as depicted in Table 1. Penile hemodynamic parameters (PSV, EDF, and RI) and EHS are described in Table 2. The diagnosis of arterial insufficiency and veno-occlusive dysfunction was made in 3 (4.34%) and 4 (5.79%) patients, respectively. Only 2 patients needed alprostadil redosing to achieve their best-quality erection. None of the patients had priapism. At the beginning of the study, overall patients had a median IIEF-5 of 9 (IQR, 9-14,5), with a value of 15 (IQR, 11-22) at 5 years.

**Table 1:** Patient characteristics.

Age, y	58.47 ± 10.39
Body mass index, kg/m^2^	28.73 ± 3.36
Marital status	
Married	69.6 (48)
Divorced	11.5 (8)
Single	11.5 (8)
Widowed	7.2 (5)
Arterial hypertension	47.8 (33)
Dyslipidemia	46.4 (32)
Active or past smoking	39.1 (27)
Diabetes	36.2 (25)
History of stroke	10.1 (7)
Coronary artery disease	7.2 (5)
Chronic kidney insufficiency	2.9 (2)
Peripheral artery disease	2.9 (2)

Values are mean ± SD or % (*n*).

**Table 2:** Patient’s Doppler parameters and EHS at baseline evaluation.

PDU	
PSV, mL/s	57.27 ± 18.62
EDF, mL/s	−0.97 ± 6.50
RI	0.97 ± 0.12
EHS	
I	2.9% (2)
II	26.1% (18)
III	33.3% (23)
IV	37.7% (26)

Values are mean ± SD or % (*n*).

Abbreviations: EDF = end-diastolic flow; EHS = Erection Hardness Score; PDU = penile duplex ultrasound; PSV = peak systolic velocity; RI = resistive index.

Of all available men, 8 (11.50%) were unable to continue sexual activity due to medical reasons, but none of them had abnormal PDU or IIT. Of all patients who were initially started on 100 mg of sildenafil citrate as per protocol, 4 (5.80%) had switched to intraurethral alprostadil and 3 (4.35%) to intracavernous alprostadil as shown in Table 3. Five (7.24%) patients had a PP implanted and 8 (11.59%) had failed first and second-line treatments but refused PP implantation, totaling 13 (18.83%) patients meeting the study definition of refractory ED.

**Table 3:** Therapeutic status for erectile dysfunction at 5 years of follow-up for patients started on PDE5 inhibitors.

	**PDE5 inhibitor**	**Intraurethral alprostadil**	**Intracavernous alprostadil**	**Penile prothesis**
**Maintain treatment with satisfaction**	58.0 (40)	2.90 (2)	2.90 (2)	10.1 (7)
**Cessation**				
**Lack effect**	15.9 (11)	2.90 (2)		
** Ceased sexual activity**	11.6 (8)			
** Secondary effects**	1.4 (1)			
** Financial reasons**	1.4 (1)			
** Medical comorbidity**	11.6 (8)			
** Partner’s refusal**			1.4 (1)	

Values are % (*n*).

Abbreviation: PDE5 = phosphodiesterase type 5.

**Figure 1:**
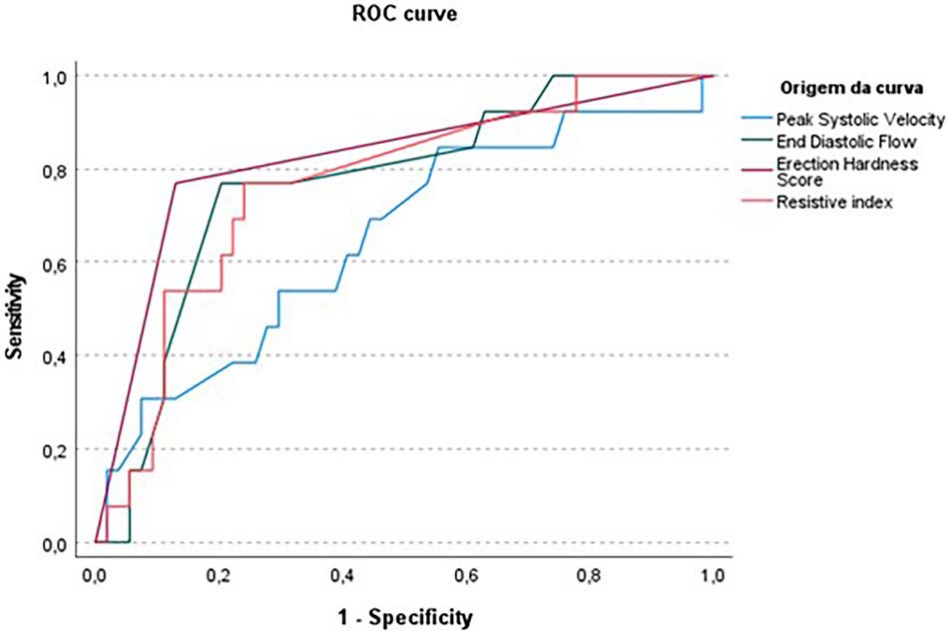
Receiver-operating characteristic curves for Doppler ultrasound measurements and Erection Hardness Score predicting refractory erectile dysfunction.

The ROC curves for PSV, EDF, RI, and EHS for refractory ED are shown in Figure 1. The AUCs for EHS, PSV, EDF, and RI to discriminate refractory ED were 0.820 (95% CI, 0.687-0.899), 0.613 (95% CI, 0.486-0.729), 0.730 (95% CI, 0.608-0.831), and 0.714 (95% CI, 0.590-0.818), respectively. EHS was classified as excellent, PSV as acceptable, and EDF and RI as good in discriminate refractory ED.

Pairwise comparison of ROC curves showed a statistically significant difference between EHS and PSV (*P* = .0464), as shown in Table 4.

**Table 4:** Comparison between the AUCs for Doppler measurements and Erection Hardness Score for predicting refractory erectile dysfunction.

	*P* value
PSV – EDF	.3597
PSV – RI	.3728
PSV – EHS	.0464
EDF – RI	.5859
EHS – RI	.1393
EHS – EDF	.2766

Abbreviations: AUC = area under the curve; EDF = end-diastolic flow; EHS = Erection Hardness Score; PSV = peak systolic velocity; RI = resistive index.

An abnormal EHS had a higher sensitivity and specificity when compared with PSV, EDF, and RI, as shown in Table 5.

**Table 5:** Sensitivity and specificity commonly utilized cutoffs for Doppler measurements and EHS in predicting refractory erectile dysfunction.

	Sensitivity (%)	Specificity (%)
PSV <30 cm/s	23.1	90.7
EDF >3 cm/s	69.2	72.2
RI <0.8	15.4	94.4
EHS ≤2	76.9	89.4

Abbreviations: EDF = end-diastolic flow; EHS = Erection Hardness Score; PSV = peak systolic velocity; RI = resistive index.

## Discussion

The European Association of Urology and the American Urological Association have both endorsed the use of PDU as a specific diagnostic test in ED.^7^^,^^21^ However, it requires skill and costly hardware to be performed. Our study intended to assess the added value of common PDU measurements to the often described as “limited” IIT in predicting refractory ED.

More than one-third of ED patients are poor responders to oral therapy,^13–15^ and in our sample almost 40% discontinued treatment, as seen in Table 3. It is important to identify these patients as early as possible to give the best treatment option according to natural history of that ED, gain the patient’s trust by explaining the possibilities, and prepare them for the possibility of a PP.

Our group pragmatically defined refractory ED as the need to have a PP implanted to treat ED, as this is an important subgroup of patients that needs to be recognized early on and as a stepwise treatment approach will often delay a satisfactory treatment and leave patients feeling anxious and frustrated over their failures with nonsurgical treatments until a PP is finally discussed.

Although PDU is not the gold-standard, it is well established and used as an etiological discriminator, namely labeling patients with a vasculogenic diagnosis. However, the value of that label depends on its power to predict those who will not respond to nonsurgical treatment, and will often need a PP (the so-called difficult-to-treat/refractory ED). This power to predict is not well established yet, and these labels are rather used as a pretext to implant or deny a PP to a patient seeking care for ED.

In the present study, an abnormal EHS was an excellent predictor for refractory ED at 5-year follow-up, as shown by its AUC, sensitivity, and specificity. The EHS was developed by Goldstein et al^22^ for use in the clinical trials of sildenafil citrate to provide an assessment of clinical efficacy. The EHS was described as a good predictor of treatment response and satisfaction with sildenafil citrate, even surpassing or showing no difference to common PDU measured Doppler parameters.

The importance of erection hardness has been explained by several authors. Achieving it is associated with a cascade of positive psychosocial events, leading to overall sexual satisfaction for both patient and partner.^10^ For some, it is the definition of masculinity and 4 of the 5 items on the Sexual Health Inventory for Men and questions 2, 4, 5, and 15 of the IIEF focus on hardness, reflecting its importance.^23^^,^^24^ Erection rigidity gives a picture of the physiological state of penile erection, combining the effect of both arteries and veins, as well as the subjective assessment of the “eyes of the beholder.” In that sense, the EHS can be a measuring tool that translates a physiologic response and its subjective interpretation. In opposition, PSV and EDF are single measures and RI translates peripheral resistance to blood flow and that is not obtained directly, but rather is obtained by a formula using PSV and EDF. PDU is also time-consuming and requires both hardware and ultrasound skills, while the EHS is a simple, reproducible, less user-dependent tool in which the patient can give their subjective input. That is, formal interpretation may show that rigidity is insufficient for penetration, but the patient may be comfortable and obtain sexual satisfaction with it, and it is this discrepancy that our group believes is the added value of IIT that PDU measurements cannot match. In that sense, having abnormal PDU parameters may not be so reliable in predicting patients that will have refractory ED, and a perceived normal erection during IIT despite objectively abnormal PDU parameters may be a better predictor.

In the present study, from the 13 patients with refractory ED, an abnormal EHS could have predicted 10, while PDU parameters (combining diagnosis of arterial insufficiency and veno-occlusive dysfunction) failed to predict refractory ED in 8 patients, showing the superiority EHS over PDU.

There are certain limitations in our study. When compared with other studies in this topic, our sample size can be considered small, and the number of events observed was also low. Also, it results from a single tertiary academic center, which may limit the generalizability of its conclusions. Compared with the PDU, the main limitation of the EHS is the lack of ability to identify an etiology. In a population of this age group and comorbidity, we expected more hemodynamic abnormalities on PDU. Ultimately, comparing EHS and PDU parameters relies in a patient self-reported classification (thus, subjective), while the other is a hemodynamic parameter (thus, objective).

However, to our knowledge, this is the first prospective study to compare the EHS during IIT with a PDU in the long term for their ability to predict which patients presenting for ED will have poor treatment response and will need to implant a PP. All the patients were observed by experienced urologists with specialized training in sexual medicine and, to avoid suggestion bias, the PDU was conducted by a blinded third party who followed a standardized protocol and was unaware of the study objectives. Nevertheless, treating physicians were not blinded to the PDU or EHS results. Moreover, a very low loss to follow-up was observed (only 3.89%), which renders an attrition bias unlikely. In reality, the EHS after IIT is properly part of PDU, so the patients were not submitted to additional burden.

## Conclusion

Based on our work, the data suggest that an abnormal EHS during an IIT is, at least, noninferior than an abnormal PDU in predicting those patients that will not respond to nonsurgical treatments and that will need a PP in long term. Larger studies are needed to confirm our results.

## Funding

None declared.

*Conflicts of interest:* None declared.
